# Activation of Delta-Opioid Receptor Protects ARPE19 Cells against Oxygen-Glucose Deprivation/Reoxygenation-Induced Necroptosis and Apoptosis by Inhibiting the Release of TNF-*α*

**DOI:** 10.1155/2022/2285663

**Published:** 2022-11-19

**Authors:** Runjie Guo, Ping Chen, Tiantian Fu, Ren Zhang, Yuan Zhu, Nange Jin, Hong Xu, Yong Xia, Xuesong Tian

**Affiliations:** ^1^Experiment Center of Science and Technology, Laboratory Animal Center, Shanghai University of Traditional Chinese Medicine, Shanghai 201203, China; ^2^Shanghai Chinese Medicine Literature Museum, Shanghai 201203, China; ^3^Shanghai Jinshan District Hospital of Traditional Chinese and Western Medicine, Shanghai 201501, China; ^4^Department of Vision Sciences, University of Houston College of Optometry, Houston, TX 77204, USA; ^5^Department of Acupuncture-Moxibustion, Longhua Hospital, Shanghai University of Traditional Chinese Medicine, Shanghai 201203, China; ^6^School of Acupuncture-Moxibustion and Tuina, Shanghai University of Traditional Chinese Medicine, Shanghai 201203, China

## Abstract

**Purpose:**

Retinal ischemia–reperfusion injury (RIRI) is the basis of the pathology that leads to many retinal diseases and induces necroptosis and apoptosis. Tumor necrosis factor-*α* (TNF-*α*) is critically involved in necroptosis and apoptosis. Delta-opioid receptor (DOR) activation inhibits TNF-*α* release in our previous studies, it might prevent necroptosis and apoptosis by inhibiting the release of TNF-*α*. However, the role of TNF-*α* and DOR in necroptosis and apoptosis of retinal pigment epithelial (RPE) cells remains largely unknown. Here, we explored the mechanisms of TNF-*α* and DOR in necroptosis and apoptosis using an oxygen-glucose deprivation/reoxygenation (OGD/R) model of adult retinal pigment epithelial cell line-19 (ARPE19) cells.

**Materials and Methods:**

ARPE19 cells were exposed to OGD/R conditions to mimic RIRI in vitro. Cell viability was quantified using the Cell Counting Kit-8 (CCK-8) assay. Morphological changes were observed by inverted microscopy. TNF-*α* protein levels in cell lysates were measured by enzyme-linked immunosorbent assay (ELISA). The DOR agonist TAN-67 and antagonist naltrindole (NTI) were used to pretreat cells for 1 or 2 hours before OGD24/R36 administration. Calcein acetoxymethylester/propidium iodide (Calcein-AM/PI) and Terminal deoxynucleotidyl transferase dUTP nick-end labeling (TUNEL) staining were used to detect necroptotic and apoptotic ARPE19 cells, respectively. The protein expression of DOR, p-RIP1 (RIP1), p-RIP3 (RIP3), p-MLKL (MLKL), and cleaved Caspase3 (Caspase3) was measured by western blotting.

**Results:**

OGD severely damaged ARPE19 cells. Prolonged reoxygenation significantly increased TNF-*α* level and decreased DOR expression in ARPE19 cells. Pretreatment with the DOR agonist TAN-67 (10 *µ*M) significantly improved ARPE19 cell viability after OGD24/R36 by reducing the number of necroptotic and apoptotic cells. Furthermore, DOR activation significantly inhibited TNF-*α* release and suppressed the expression of proteins related to necroptosis and apoptosis, including p-RIP1, p-RIP3, p-MLKL, and cleaved Caspase3, after OGD24/R36. This effect was reversed by the DOR antagonist NTI.

**Conclusion:**

These results strongly suggest that DOR activation inhibits necroptosis and apoptosis by decreasing TNF-*α* release, leading to the prevention of OGD/R-induced injury in ARPE19 cells. This study provides an innovative idea for clinical treatment strategies for retinal damage and vision loss due to RIRI.

## 1. Introduction

Retinal ischemia–reperfusion injury (RIRI) is the basis of the pathology that leads to progressive visual loss and blindness in many retinal diseases, such as glaucoma [1], diabetic retinopathy [2], and retinal vascular occlusion disease [3]. The pathogenesis of RIRI is complex, and there are currently four main types of injury mechanisms: inflammatory reactions, cellular death, excitatory amino acids, and reactive oxygen species [4, 5]. Among them, cellular death is the key to impairments in vision and retinal function [6, 7]. Retinal pigment epithelial (RPE) cells are located between Bruch's membrane of the choroid and the retinal neurepithelium and perform a variety of functions, such as transporting nutrients from the choroid to photoreceptors, expelling metabolic waste, engulfing detached photoreceptor outer segments, and maintaining the stability of retinal appendages by removing fluid from the retinal sites of the inner photoreceptor layer [8–10]. RPE cell death occurs in RIRI, ultimately causing progressive and irreversible degeneration of retinal ganglion cells and vision loss [11]. Thus, protecting RPE cells can be considered an effective strategy to prevent the devastating damage of RIRI events.

RIRI alters neuronal tissue function by inducing neuronal cell death [12]. In RIRI, RPE cells mainly undergo two types of programmed cell death, necroptosis, and apoptosis, and these two types of death do not exist in isolation. They often occur together or one after the other [7]. Tumor necrosis factor-*α* (TNF-*α*) is well known to be the major trigger of necroptosis and apoptosis [13]. When TNF-*α* binds to the TNF receptor on the cell membrane, it further activates the downstream necroptosis and apoptosis pathways [14]. When receptor-interacting protein kinase 1 (RIP1) is activated, it can recruit receptor-interacting protein kinase 3 (RIP3), causing RIP3 phosphorylation. Once RIP3 is activated, mixed lineage kinase domain-like protein (MLKL) is assembled and phosphorylated. Eventually, necrosomes are formed, which triggers necroptosis. Apoptosis mainly occurs through the activation of Caspase 8 via the apoptotic pathway, which further activates Caspase 3 (cleaved Caspase 3) to cause apoptosis [15, 16]. Therefore, inhibiting TNF-*α* is particularly important for reducing necroptosis and cell apoptosis.

The delta-opioid receptor (DOR) is an oxygen-sensitive protein sensitive to ischemia and hypoxia [17]. Several experiments in vivo and in vitro support the essential role of DOR in the fight against ischemic and hypoxic injury [18, 19]. Furthermore, it has been reported that DOR is widely distributed in the retina and plays an important role in protecting the retina [20]. Peng et al. demonstrated that in RIRI, the upregulation of DOR expression corrected the redox imbalance in the impaired retina and had a protective effect on the retina [21]. In addition, previous studies in our laboratory have confirmed that DOR activation can inhibit the increase in TNF-*α* caused by cerebral ischemia–reperfusion and protect neurons from death in vivo and in vitro [22]. As the retina is an extension of the central nervous system, whether DOR activation in the retina could also play a similar protective role in inhibiting necroptosis and apoptosis in RPE cells induced by RIRI has not been examined.

The oxygen-glucose deprivation/reoxygenation (OGD/R) cell model can successfully mimic the acute restriction of metabolites and oxygen supply during ischemia–reperfusion injury [23]. Thus, (1) OGD/R model was used to simulate RIRI in adult retinal pigment epithelial cell line-19 (ARPE19) cells; (2) agonists or antagonists of DOR were administered to examine whether DOR activation could inhibit OGD/R-induced necroptosis and apoptosis by inhibiting the release of TNF-*α* in ARPE19 cells and exploring the mechanism.

## 2. Materials and Methods

### 2.1. Cell Culture

ARPE19 cells were purchased from the National Collection of Authenticated Cell Cultures (Shanghai, China) and maintained in DMEM/F12 (Gibco, USA) containing 10% fetal bovine serum (FBS) (Gibco, USA) and 1% penicillin/streptomycin (Beyotime, China) at 37°C with 5% CO_2_. The medium was changed every other day and regularly tested for mycoplasma contamination. Cells in the logarithmic growth phase were used for subsequent experiments.

### 2.2. Cell Culture Treatment and OGD/R

Cells in the logarithmic growth stage were randomly assigned to five groups and subjected to different treatments as follows: (1) control group; (2) OGD/R group; (3) agonist group (Tan-67 + OGD/R); (4) antagonist group (naltrindole (NTI) + OGD/R); and (5) coadministration group (NTI + Tan-67 + OGD/R). TAN-67 and NTI were dissolved in phosphate-buffered saline(PBS) (pH 7.2) as stock solutions (10 mM and 100 mM for each) and stored at −20°C. Immediately before use, the stock was quickly thawed and diluted to the appropriate concentration with a normal culture medium. In the agonist and antagonist groups, ARPE19 cells were pretreated with TAN-67 or NTI alone for one hour. In comparison, cells in the coadministration group were incubated with NTI for one hour before TAN-67 was added for one hour followed by OGD/R.

OGD/R was used as an in vitro model of ischemia–reperfusion injury. First, ARPE19 cells were washed twice with PBS (HyClone, USA) and cultured in DMEM without glucose and FBS in a hypoxia chamber that was flushed with a 95% N_2_/5% CO_2_ gas mixture at 3 L/min for 15 min at room temperature. Then, the chamber was placed in a thermostatic incubator (Thermo Fisher Scientific, USA) for oxygen and glucose deprivation. After OGD, the cells were returned to normal DMEM/F12 under normoxic conditions and underwent reoxygenation. Cells in the control group were cultured under normal conditions for the corresponding times.

### 2.3. Analysis of Cell Viability

Cell viability was monitored with a Cell Counting Kit-8 (CCK-8) assay (Dojindo, Japan) according to the manufacturer's suggestions. Briefly, 100 *μ*l of media containing 5 × 10^3^ ARPE19 cells were seeded in 96-well plates. Following modeling, 10 *μ*l of CCK-8 solution was added to each well, and the plates were placed in a thermostatic incubator for half an hour and then assayed using a microplate reader (Thermo Fisher Scientific, USA) at a wavelength of 450 nm. Cell viability is expressed as the percentage of cell viability compared to the control group.

### 2.4. Measurement of TNF-*α* Protein Levels

TNF-*α* levels in cell lysates were measured by anti-human TNF-*α*enzyme-linked immunosorbent assay (ELISA) (R&D Systems, USA) according to the manufacturer's instructions. In brief, protein samples were extracted from cells by sonication. The samples (100 *μ*L/well) were added to 96-well plates and incubated at 37°C for 90 minutes. After the samples were washed with washing buffer three times, HRP (100 *μ*L/well) was added to the wells and incubated at 37°C for 30 min. Then, the wells were washed three times with washing buffer and developed with TMB (100 *μ*L/well) for 15 min. After the termination solution was added, the absorbance was measured at 450 nm with a microplate reader. The concentration of TNF-*α* was calculated by a TNF-*α* standard curve.

### 2.5. Western Blot Analysis

Western blotting was performed according to a standard method, as described previously [22]. The following primary antibodies were used in this study: RIP1 (1 : 2000, cell signaling, 45726, USA), p-RIP1 (1 : 2000, cell signaling, 53286, USA), RIP3 (1 : 2000, Abcam, ab226297, USA), p-RIP3 (1 : 2000, Abcam, ab195117, USA), MLKL (1 : 2000, Abcam, ab243142, USA), p-MLKL (1 : 2000, Abcam, ab187091, USA), Caspase3 (1 : 2000, cell signaling, 9662, USA), cleaved Caspase3 (1 : 2000, cell signaling, 9664, USA), DOR (1 : 2000, Millipore, AB1560, USA), and *β*-actin (1 : 2000, Sigma, A5441, USA). Goat anti-rabbit IgG (1 : 4000) and goat anti-mouse IgG (1 : 4000) were purchased from Sigma. The protein bands were quantified by densitometry using ImageJ software, and protein expression was normalized to the expression of the internal control.

### 2.6. Calcein-AM and PI Staining

Viable or necroptotic ARPE19 cells were stained with calcein acetoxymethylester (Calcein-AM) or propidium iodide (PI) according to the kit instructions (Beyotime Biotechnology, China). The primary steps are briefly described. The culture medium was discarded, and the wells were washed twice with PBS. The dye solution was composed of Calcein-AM, PI, and detection buffer at a volume ratio of 1 : 1:1000. It was added to six-well plates and incubated at 37°C for 30 min in the dark. At the end of the incubation, the wells were washed twice with PBS, and Hoechst 33258 was added and incubated at 37°C for 10 min in the dark. Finally, images were taken by fluorescence microscopy (Nikon, Japan). The number of PI-positive cells was analyzed using ImageJ software.

### 2.7. TUNEL Staining

ARPE19 cell apoptosis was determined using an in situ terminal deoxynucleotidyl transferase dUTP nick-end labeling nick-end labeling (TUNEL) assay (Beyotime Biotechnology, China). The specific processes were performed according to the instructions. The cells were fixed in 4% paraformaldehyde for 30 min at room temperature. After being washed three times with PBS, the cells were permeabilized with 0.3% Triton X-100 in PBS for 5 min at room temperature. The enzyme solution (TdT) and labeling solution (dUTP) were mixed with the TUNEL detection solution at a dilution of 1 : 9. TUNEL detection solution was added to each well and incubated at 37°C in the dark for 1 h. Before detection, the wells were washed twice with PBS, and Hoechst 33258 was added and incubated at 37°C for 10 min in the dark. At the end of the incubation, images were obtained by fluorescence microscopy, and the analysis was performed using ImageJ software.

### 2.8. Data and Statistical Analysis

The data are presented as the mean ± SEM and analyzed by Student's *t*-tests or one-way ANOVA followed by post hoc comparisons with Dunnett's post hoc test and Fisher's LSD test. Statistical analysis was conducted using SPSS software (version 23; IBM Corp., Armonk, NY, USA). A *P* value <0.05 was considered statistically significant.

## 3. Results

### 3.1. Different Durations of Oxygen and Glucose Deprivation Have Different Effects on ARPE19 Cell Viability and Morphology

**Figure 1:**
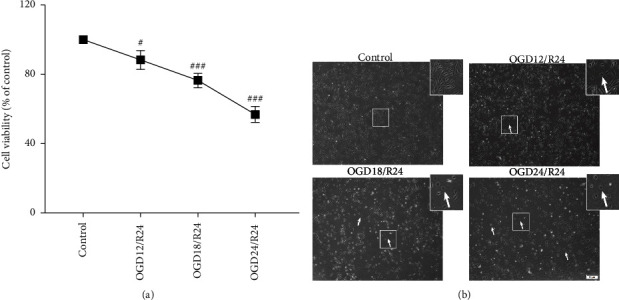
Different durations of oxygen and glucose deprivation have diverse effects on ARPE19 cell viability and morphology. (a) Cell viability was determined by a cell counting kit-8 assay. The results are expressed as proportions of surviving cells compared with the control (*n* = 5 per group). (b) Morphological changes in ARPE19 cells were observed by inverted microscopy. White arrows indicate severely shrunken and round cells (*n* = 3 per group). Data are presented as means ± SEM, and group differences were analyzed by Student's *t*-tests. ^#^*P* < 0.05, ^###^*P* < 0.001 vs. Control. Scale bar = 50 *μ*m.

To construct a stable OGD/R model in ARPE19 cells, the different duration and extents of OGD/R (12 h, 18 h, and 24 h) were examined. We fixed the reoxygenation time at 24 h. Then, CCK-8 and inverted microscopy were used to examine cellular viability and morphological changes, respectively. As shown in Figure 1(a), cell viability gradually decreased with prolonged oxygen and glucose deprivation time. Compared with that in the control group, cell viability decreased to approximately 85% in the OGD12/R24 group (^#^*P* < 0.05), 78% in the OGD18/R24 group (^###^*P* < 0.001), and 60% in the OGD24/R24 group (^###^*P* < 0.001), respectively. As shown in Figure 1(b), the morphology of ARPE19 cells in the control group was fusiform, and the cells aggregated in a colony form. With increasing oxygen and glucose deprivation durations, floating cells with spherical or round shapes (white arrows) and cellular debris, probably from dead cells, increased. Few cells remained attached to the cell culture plate, while most cells were rounded and free-floating in the OGD24/R24 group.

These results demonstrate that various durations of oxygen-glucose deprivation caused different degrees of injury in ARPE19 cells. Moreover, OGD24/R24 markedly decreased cell viability to approximately 60% and resulted in considerable alterations in cell morphology, consistent with the injury conditions of OGD/R modeling.

### 3.2. Different Durations of Reoxygenation Have Different Effects on DOR and TNF-*α* Protein Levels in ARPE19 Cells

**Figure 2:**
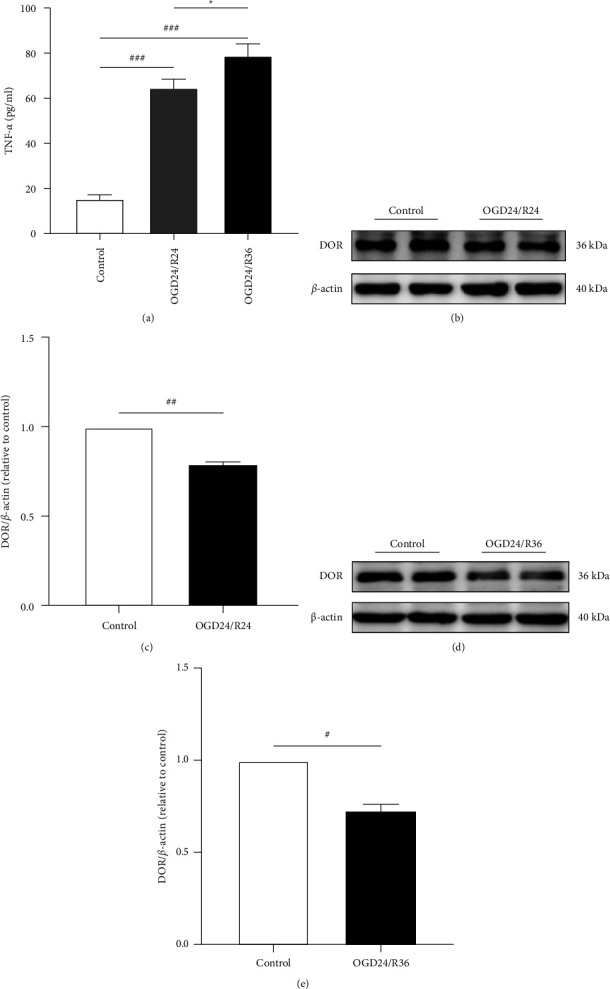
Different durations of reoxygenation have diverse effects on DOR and TNF-*α* protein levels in ARPE19 cells. (a) TNF-*α* levels in cell lysates after different reoxygenation times were measured by ELISA (*n* = 5 per group). (b–e) The protein expression of DOR after different reoxygenation times was examined by Western blotting (*n* = 3 per group). Quantification of the density values of DOR was normalized to those of *β*-actin. Data are presented as means ± SEM, and group differences were analyzed by Student's *t*-tests. ^#^*P* < 0.05, ^##^*P* < 0.01, ^###^*P* < 0.001, compared with the control group; ^*∗*^*P* < 0.05, OGD24/R24 group vs. OGD24/R36 group.

It has been reported that different reoxygenation time points also had diverse effects on cells in an OGD/R model [24, 25]. Thus, we examined the TNF-*α* and DOR protein levels in ARPE19 cells to determine whether various durations of reoxygenation have different effects on ARPE19 cells. TNF-*α* protein levels in cell lysates were measured by ELISA (Figure 2(a)), and the levels in the control group were extremely low. After OGD/R, the levels of TNF-α increased significantly (^###^*P* < 0.001); moreover, the TNF-α levels in the OGD24/R36 group were significantly higher than those in the OGD24/R24 group (^*∗*^*P* < 0.05). Western blotting showed abundant DOR expression in the control group; however, DOR expression showed a relative decrease after OGD/R (Figures 2(b), 2(d)). The protein expression of DOR in the OGD24/R24 group and OGD24/R36 group decreased by approximately 30% and 40% compared with that in the control group (Figures 2(c), 2(e)).

We can conclude that under fixed oxygen and glucose deprivation times, different reoxygenation times have distinct effects on ARPE19 cells. In the OGD24/R36 group, TNF-α protein levels increased the most, and DOR protein expression decreased the most. Here the OGD24/R36 group was used as the modeling condition for subsequent experiments.

### 3.3. The Effects of the DOR Agonist TAN-67 and the Antagonist NTI on the Viability of ARPE19 Cells Induced by OGD24/R36

**Figure 3:**
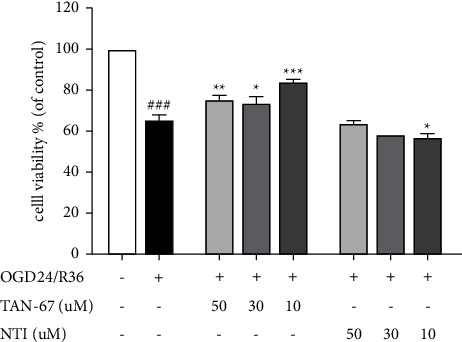
The effects of the DOR agonist TAN-67 and the antagonist NTI on the viability of ARPE19 cells induced by OGD24/R36. The viability of ARPE19 cells that were pretreated with the DOR agonist TAN-67 (50, 30, 10 *μ*M) or the antagonist NTI (50, 30, 10 *μ*M) and stimulated with or without OGD24/R36 was determined by a cell counting kit-8 assay. (+ or − indicates with or without OGD24/R36/TAN-67/NTI) The results are expressed as proportions of surviving cells compared with the controls (*n* = 5 per group). Data are presented as means ± SEM, and group differences were analyzed by one-way ANOVA with Dunnett's post hoc test. ^###^*P* < 0.001, compared with the control group; ^*∗*^*P* < 0.05, ^*∗∗*^*P* < 0.01, ^*∗∗∗*^*P* < 0.001, compared with the OGD24/R36 group.

To examine the impacts of DOR protein activation and antagonism on the cell viability after OGD24/R36, ARPE19 cells were pretreated with different concentrations of the DOR agonist TAN-67 (50, 30, 10 *µ*M) or the antagonist NTI (50, 30, 10 *µ*M) for one hour. CCK-8 was used to examine cellular viability after OGD24/R36 (Figure 3). The results showed that OGD24/R36 resulted in a significant decline in cell viability (^###^*P* < 0.001). Pretreatment with the DOR agonist TAN-67 increased cell viability compared with that in the OGD24/R36 group (^*∗*^*P* < 0.05, ^*∗∗*^*P* < 0.01, ^*∗∗∗*^*P* < 0.001). Among them, 10 *µ*M TAN-67 had the best effect on increasing the viability of ARPE19 cells. Treatment with 10 *μ*M NTI resulted in a substantial decrease in cell viability (^*∗*^*P* < 0.05), while no obvious changes were observed when the cells were treated with 50 *μ*M and 30 *μ*M NTI compared with that in the OGD24/R36 group. These results suggested that DOR activation could improve the survival of ARPE19 cells, and the opposite effects were observed in response to DOR inhibition.

### 3.4. The DOR Agonist TAN-67 Reduced the Number of Necroptotic and Apoptotic ARPE19 Cells after OGD24/R36

**Figure 4:**
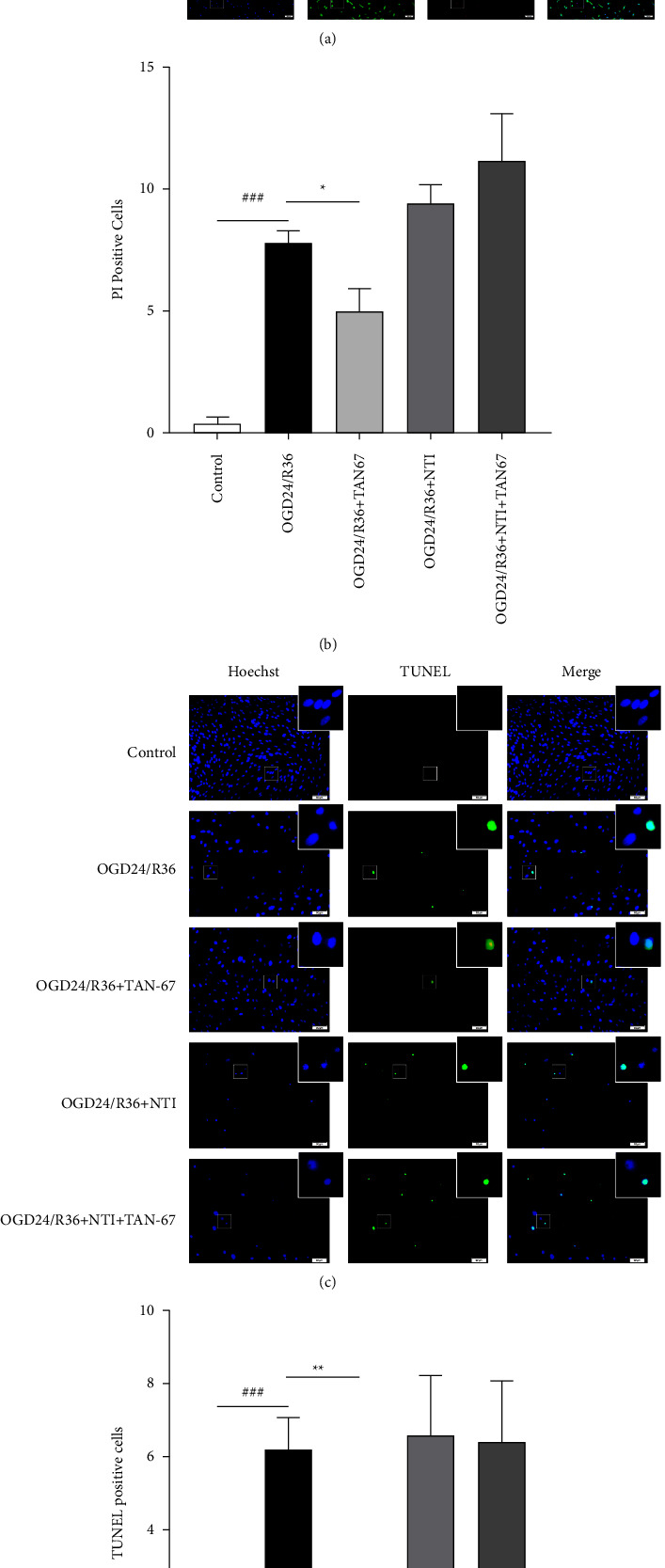
The DOR agonist TAN-67 reduced the number of necroptotic and apoptotic ARPE19 cells after OGD24/R36. (a) A Calcein-AM/PI kit was used to distinguish live cells from necroptotic cells. Representative images from left to right: nuclear staining with Hoechst 33258 (blue); calcein-AM staining of live cells (green); PI staining of necroptotic cells (red); merged image. The white square indicates representative cells that are magnified. Scale bar = 100 *μ*m. (b) The bar graph represents the number of PI-positive cells in each group (*n* = 5 per group). (c) Apoptosis was determined using a TUNEL kit. Representative images from left to right: nuclear staining with Hoechst 33258 (blue); TUNEL of apoptotic cells (green); merged image. The white square indicates representative cells that are magnified. Scale bar = 50 *μ*m. (d) The bar graph represents the number of TUNEL-positive cells in each group (*n* = 5 per group). Data are presented as means ± SEM, and group differences were analyzed by one-way ANOVA with Fisher's LSD test. ^###^*P* < 0.001 compared with the control group; *∗P* < 0.05, *∗∗P* <  0.01 compared with the OGD24/R36 group.

We further examined whether the DOR agonist TAN-67 could inhibit necroptosis and apoptosis in ARPE19 cells after OGD24/R36. Calcein-AM/PI and TUNEL staining were used to examine necroptosis and apoptosis in ARPE19 cells, respectively. As shown in Figure 4(a), Calcein-AM staining (green) represents live cells, while PI staining (red) represents necroptotic cells. Based on the results shown in Figure 4(b), after OGD24/R36, a marked increase in PI-positive cells was observed in the OGD24/R36 group (^##^*P* < 0.001), while the number of PI-positive cells was lower in the group that was treated with TNA-67 than in the OGD24/R36 group (^*∗*^*P* < 0.05). In the DOR antagonist NTI treatment group, no significant changes in the numbers of PI-positive cells were observed compared with those in the OGD24/R36 group. However, NTI substantially eliminated the effects of TAN-67 in the co-administration group, and there were no obvious changes in the number of PI-positive cells compared with that in the OGD24/R36 group. The TUNEL staining results are shown in Figure 4(c). Green fluorescence represents apoptotic cells stained by TUNEL, and the number of apoptotic cells was consistent with PI staining results. Compared with that in the control group, the number of TUNEL-positive cells significantly increased in the OGD24/R36 group (^###^*P* < 0.001). The TAN-67 treatment group showed a dramatic decline in TUNEL-positive cells (^*∗*^*P* < 0.05), while the effect of TAN-67 (10 M) was blocked by concomitant incubation with NTI.

Taken together, these results suggest that the DOR agonist TAN-67 reduces the number of necroptotic and apoptotic ARPE19 cells after OGD24/R36.

### 3.5. The DOR Agonist TAN-67 Suppressed TNF-*α* Levels and the Expression of Necroptotic and Apoptotic Proteins in ARPE19 Cells Induced by OGD24/R36

**Figure 5:**
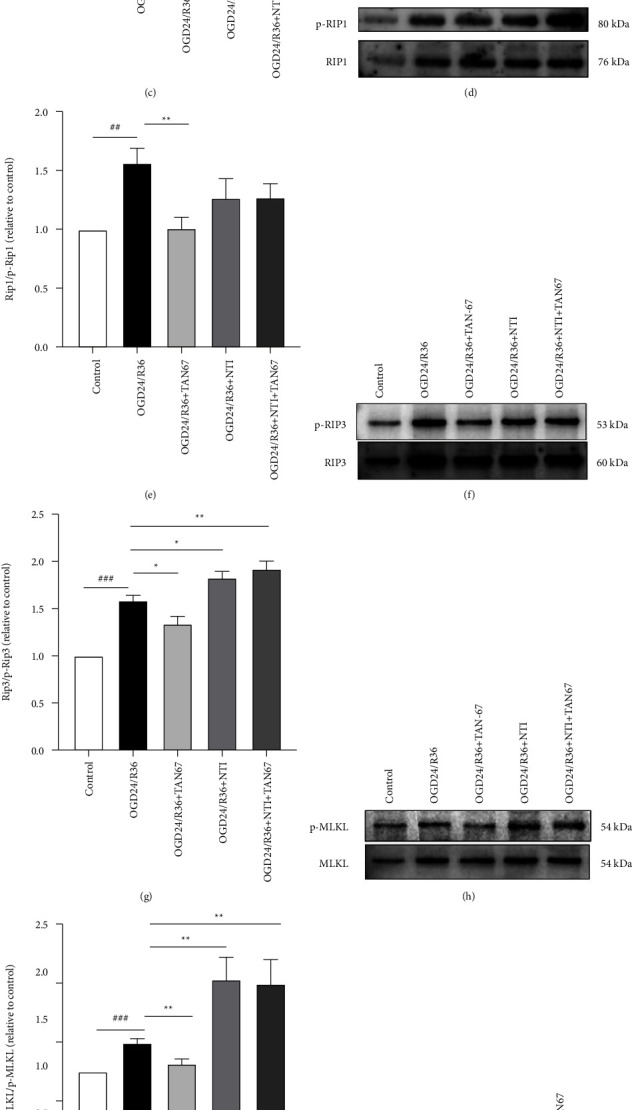
The DOR agonist TAN-67 suppresses TNF-*α* levels and the expression of necroptotic and apoptotic proteins in ARPE19 cells induced by OGD24/R36. (a) TNF-*α* levels in cell lysates from each group were measured by ELISA (*n* = 5 per group). (b–c) The level of DOR expression was examined by Western blotting, and the density values were normalized to the levels of *β*-actin (*n* = 3 per group). (d–k) Western blot showing the activation or total abundance of p-RIP1 (RIP1), p-RIP3 (RIP3), p-MLKL (MLKL), and cleaved Caspase-3 (Caspase-3) as indicated (*n* = 3 per group). P-RIP1, phosphorylated RIP1; p-RIP3, phosphorylated RIP3; p-MLKL, phosphorylated MLKL. Data are presented as means ± SEM, and group differences were analyzed by one-way ANOVA with Dunnett's post hoc test. ^##^*P* < 0.01, ^###^*P* < 0.001 compared with the control group; *∗P* < 0.05, *∗∗P* < 0.01, *∗∗∗P* < 0.01, compared with the OGD24/R36 group.

To test whether the key cytokine TNF-*α* is involved in the mechanism triggering necroptosis and apoptosis, ELISA was performed to examine the levels of TNF-*α* in ARPE19 cells. As shown in Figure 5(a) the levels of TNF-*α* in the OGD24/R36 group were increased compared with those in the control group (^###^*P* < 0.001). Pretreatment with TAN-67 inhibited the OGD24/R36-induced increase in TNF-*α* (^*∗*^*P* < 0.05), but this effect was eliminated by NTI pretreatment. NTI pretreatment alone also showed no significant effects compared with the OGD24/R36 group.

By activating DOR, we evaluated whether TAN-67 inhibits OGD24/R36-induced necroptosis and apoptosis in ARPE19 cells. The protein expression of DOR, p-RIP1, p-RIP3, p-MLKL, and cleaved Caspase3 in ARPE19 cells was examined by Western blotting (Figures 5(b)–5(k)). Compared with the control, OGD24/R36 resulted in marked increases in p-RIP1, p-RIP3, p-MLKL, and cleaved Caspase3 expression levels, while DOR expression was reduced (^##^*P* < 0.01, ^###^*P* < 0.001). After pretreatment with TAN-67, DOR was activated in ARPE19 cells, and its expression was increased; in contrast, the levels of p-RIP1, p-RIP3, p-MLKL, and cleaved Caspase3 were significantly diminished compared with those in the OGD24/R36 group (^*∗*^*P* < 0.05, ^*∗∗*^*P* < 0.01, ^*∗∗∗*^*P* < 0.001). However, NTI coadministration blocked the effects of TAN-67. NTI treatment alone induced no substantial changes compared to the OGD24/R36 group.

These data suggest that TAN-67 can activate DOR and reduce the release of the inflammatory factor TNF-*α* to inhibit downstream necroptosis and the expression of the apoptosis-related proteins p-RIP1, p-RIP3, p-MLKL, and cleaved Caspase-3.

## 4. Discussion

Based on this research, the following conclusions can be drawn: (1) the viability of ARPE19 cells decreased significantly with prolonged oxygen-glucose deprivation. (2) The more extended the reperfusion time after oxygen-glucose deprivation, the more TNF-*α* and the less DOR protein expression in the cells. (3) DOR activation significantly reduces the release of TNF-*α* in ARPE19 cells after OGD24/R36, which can decrease the number of necroptotic and apoptotic cells and inhibit related protein expression from exerting a protective effect on ARPE19 cells.

OGD/R is a classic in vitro model for simulating ischemia–reperfusion injury and has been used in ischemia–reperfusion injury experiments to study the brain, heart, and kidney in vitro [26–28]. Previous studies have shown that different oxygen and glucose deprivation durations have robust impacts on cell survival [29, 30]. Therefore, we examined the duration of oxygen and glucose deprivation and the survival rates of ARPE19 cells at different time points by CCK-8 assays. The cell survival rate in response to OGD24/R24 was 60%, and most cells showed obvious damage, which was consistent with the conditions of model damage. It has also been reported that cells release more inflammatory factors with the prolongation of reperfusion. [31, 32]. We then determined the time course of changes in TNF-*α* after reoxygenation, and our results verified this point of view. The amount of TNF-*α* released at 36 hours of reperfusion was higher than that at 24 hours, and reperfusion for 36 hours induced more damage to the expression of DOR protein in ARPE19 cells. Based on the relationship between TNF-*α* and both necroptosis and apoptosis and the effect of activated DOR in ischemia–reperfusion, we chose OGD24/R36 as the optimum time for this study.

Necroptosis and apoptosis have distinctly different characteristics in terms of pathological features [33]. First, the integrity of the necroptotic cell membrane is severely damaged, the organelles are swollen and disintegrated, and chromatin in the nucleus lacks obvious morphological changes. Apoptosis is characterized by an intact cell membrane, intracellular chromosome condensation, nuclear fragmentation, and the formation of DNA bands [34]. These distinct differences can also be clearly distinguished by light microscopy, electron microscopic observation, and specific staining methods [35, 36]. In this study, calcein-AM/PI staining and TUNEL staining were used to examine necroptosis and apoptosis in ARPE19 cells, respectively. PI only penetrates damaged cell membranes and releases red fluorescence when embedded in double-stranded DNA, so it has been used to identify necroptotic cells in recent studies [37, 38]. TUNEL is a commonly used method to label apoptotic cells by specifically detecting DNA breaks that occur during apoptosis. Consistently, our results showed that the number of necroptotic and apoptotic ARPE19 cells induced by OGD24/R36 was also markedly diminished after pretreatment with the DOR agonist TAN-67, whereas the DOR antagonist NTI blocked this protective effect.

Current research suggests that TNF-*α* is a common promoter of both necroptosis and apoptosis [14]. Many previous studies, including ours, have confirmed that DOR activation can inhibit the release of TNF-*α* in cerebral ischemia–reperfusion injury and markedly attenuate the inflammatory response [22, 39]. Similarly, the results of this study demonstrated that DOR activation inhibited the ODG24/R36-induced increase in TNF-*α* levels in ARPE19 cells. The reduction in TNF-*α* may downregulate the activation of the necroptotic marker proteins p-RIP1, p-RIP3, and p-MLKL or the apoptotic marker protein cleaved Caspase3 downstream of the signaling pathway. Therefore, the DOR agonist TAN-67 reverses the OGD24/R36-induced increases in necroptosis- and apoptosis-associated proteins. Our previous studies have shown that DOR activation suppresses inflammation and is closely related to the MAPK/p38 pathway [40]. It has also been reported that TNF-*α* release is associated with the activation of the nuclear factor kappa-B (NF-*κ*B) pathway. And DOR activation can reduce TNF-*α* release by inhibiting NF-*κ*B pathway activation, thereby mitigating inflammation [41]. In the future, we will also validate the mechanisms associated with the inhibition of the inflammatory response in ARPE19 cells by agonists of DOR in OGD/R injury.

Previous studies have shown that the DOR protein is abundantly expressed in the human retinal pigment epithelium, and the administration of DOR agonists can significantly alleviate RPE cell damage and maintain the function and integrity of the retinal pigment epithelium in diabetic retinopathy [22]. As an essential component of the retinal epithelium, RPE cells play a key role in maintaining visual circulation, barrier formation, and material transport in the retina [42]. When pigment epithelial cells are injured, the retina suffers a disruption in water-electrolyte transport and a considerable accumulation of oxygen-free radicals and inflammatory factors that eventually damage the retinal ganglion cells resulting in vision loss or blindness [43]. Therefore, inhibiting RPE cell death is critical to the therapeutic and prognostic effects of retinal diseases. In the present study, DOR activation by TAN-67 dramatically affected the inhibition of necroptosis and apoptosis in ARPE19 cells after OGD/R and could decrease ARPE19 cell injury. Consistent with our recent study (data not shown), DOR activation may play a critical role in ameliorating retinal ganglion cell injury by rescuing OGD/R-induced human RPE cell damage. DOR could be a new target for the treatment of visual loss or blindness after RIRI.

## 5. Conclusion

In summary, the present study demonstrates that DOR activation can reverse the increase in TNF-*α* induced by OGD/R while alleviating TNF-*α*-induced necroptosis and apoptosis in ARPE19 cells. This finding indicates that DOR-mediated retinal protection is closely correlated with the downregulation of the TNF-*α* pathway. This study provides new insights into the clinical treatment of retinal damage and vision loss caused by RIRI.

## Data Availability

The data used to support the findings of this study are available from the corresponding author upon request.
